# Monitoring the Evolving Match Environment in Emergency Medicine 2023

**DOI:** 10.5811/westjem.18562

**Published:** 2024-12-31

**Authors:** Anthony Sielicki, Brian Milman, Andrew Little, Miriam Kulkarni, James Morris, Laura Hopson, Michael Kiemeney

**Affiliations:** * Jefferson Einstein Hospital, Department of Emergency Medicine, Philadelphia, Pennsylvania; † UT Southwestern Medical Center, Department of Emergency Medicine, Dallas, Texas; ‡ AdventHealth, Department of Emergency Medicine, Orlando, Florida; § St. John’s Riverside Hospital, Department of Emergency Medicine, Yonkers, New York; ∥ Texas Tech University Health Sciences Center, Department of Emergency Medicine, Lubbock, Texas; ** University of Michigan, Department of Emergency Medicine, Ann Arbor, Michigan; †† Loma Linda University Medical Center, Department of Emergency Medicine, Loma Linda, California

## Abstract

**Introduction:**

The 2023 National Residency Matching Program (NRMP) Match in emergency medicine (EM) left 554 spots and 132 EM programs unfilled. The Council of Residency Directors Match Task Force sought to characterize the programs that did and did not fill, learn more about their Supplemental Offer and Acceptance Program (SOAP) applicants, determine residency programs’ needs for future NRMP Matches, and inquire what actions program leaders would like to see to promote a healthy future for training in EM.

**Methods:**

We conducted a web-based survey of EM residency program leadership during March and April 2023. We generated descriptive statistics from these survey results. Thematic analysis was used for free-text responses.

**Results:**

Of 287 programs, 160 (55.7%) responded to the survey, including 59 of 132 programs (44.7%) that did not fill in the Match. Unfilled programs were overall content with the quality of applicants in the SOAP. Programs expressed varying opinions on why fewer students are choosing EM. While most agreed there are concerns about the workforce (78.1%), even more spread exists on what actions should be taken to help support the future of residency training in EM.

**Conclusion:**

Here we present data regarding the 2023 Match environment for EM and describe a residency program-level needs assessment and desire for action. Annual review of the Match data and residency program needs should be continued until we see improvement in the Match environment for EM.

Population Health Research Capsule What do we already know about this issue?

*The 2023 Match for emergency medicine left 132 residency programs unfilled and 554 unfilled spots.*

What was the research question?

*We sought to determine residency programs’ needs for future Matches and what actions program leaders desire to promote a healthy future for training in EM.*

What was the major finding of the study?

*Most respondents agreed that EM application numbers were down due to concerns about the workforce (78%), and the leading desire was to halt opening new programs (25%).*

How does this improve population health?

*These findings could improve population health by ensuring a healthy Match and training environment in EM.*

## INTRODUCTION

The National Resident Matching Program (NRMP) Match for emergency medicine (EM) has evolved over the past several years. Historically, EM has been a competitive specialty with nearly 100% program match rates.^1^ The 2022 EM Match represented a fundamental change compared to the historical data, with over 200 EM positions and over 60 residency programs unfilled.^2^ This trend continued in the 2023 match, with 554 unfilled positions across 132 programs, although this trend improved as of the 2024 Match.^3^ ^,^ ^4^ Table 1 displays several years of EM match data, which highlights the growth of residency programs, increasing number of postgraduate year-1 positions, and variable number of applicants to EM residency programs.^5^ ^,^ ^6^

**Table 1.** Emergency medicine National Resident Match Program data 2019–2024.

Year	2019	2020	2021	2022	2023	2024
# residency programs	238	256	273	277	287	292
# PGY-1 positions	2,488	2,665	2,840	2,921	3,010	3,026
# applicants	3,048	3,323	3,734	3,081	2,765	3.547
# unfilled positions (%)	30 (1.2%)	13 (0.5%)	14 (0.5%)	219 (7.5%)	554 (18.4%)	135 (4.5%)
# unfilled programs (%)	15 (6.3%)	7 (2.7%)	9 (3.3%)	69 (24.9%)	132 (46%)	54 (18.4%)

*EM*, emergency medicine; *PGY*, postgraduate year.

Several theories have been proposed to explain why fewer medical students are applying to EM. The 2030 jobs report left many concerned that there would not be enough work for all emergency physicians (EP),^7^ while others have speculated that issues with boarding, drug and nursing shortages, burnout, the COVID-19 pandemic, concerns for future novel infectious diseases, and scope of practice of non-physician medical practitioners may contribute. These issues are currently under investigation by multiple groups, including the Council of Residency Directors in EM (CORD).^8^

CORD is an organization comprised of EM residency educators and program leadership providing resources and developing best practices for education in EM. In March 2022, CORD surveyed its members to understand what the organization could do to support its members following the 2022 Match.^9^ Based on feedback from that survey, the CORD Board of Directors convened the EM Match Task Force. The primary objectives of this task force are to collect data and to intervene with regard to the increased number of unfilled EM residency positions.^10^ The initial goal of the task force was to understand the factors that led to the increased number of unfilled spots, the quality of applicants to EM, as well as interview and rank-list behaviors of programs in the 2023 Match. Additional objectives included examining residency leadership opinions on the utility of preference signaling, readiness of Supplemental Offer and Acceptance Program (SOAP) candidates, and desired actions to improve the EM Match environment.

Considering these objectives, the CORD EM Match Task Force sought to elicit the needs and perceptions from EM residency program leaders as a first step toward developing targeted interventions to improve the EM Match environment. In this paper, the members of the CORD EM Match Task Force describe results of a survey conducted following the 2023 Match.

## METHODS

The CORD EM Match Task Force members developed a survey expanding upon the work of the 2022 Murano et al survey. Consensus methodology between task force members was used to develop and refine the survey. The survey was then distributed to EM residency program leadership (program directors [PD], assistant/associate program directors [APD], clerkship directors [CD], program coordinators [PC], chairs, and general faculty members) during the CORD Academic Assembly in March 2023 in Las Vegas, NV. Survey participation was voluntary and solicited via QR code during conference sessions. The survey was also distributed on the CORD Program Director Listserv to reach program leadership who did not attend the conference. The survey was web-based and used Qualtrics (Qualtrics International Inc, Provo, UT) for data collection.

The survey collected the respondents’ residency program, their role within the program, and demographic information about the program (ie, length of training, location of program, sponsoring institutions). All program leaders were asked about the number and quality of applicants to their program as well as outcomes in the Match. For programs that did not fill and used the SOAP, we asked questions regarding the quality of applicants in the SOAP and sought feedback about the SOAP process. Additionally, all respondents were asked to identify why they thought fewer medical students are applying to EM and what additional actions they would like to see taken to improve the Match environment in EM. This study was reviewed by the Loma Linda University Institutional Review Board and given exempt status.

We analyzed data using Microsoft Excel 365 (Microsoft Corp, Redmond, WA) to calculate descriptive statistics. To avoid over-weighting perspectives from a single program, we sorted data to select a single response per program. We used the following order of consideration when more than one response was available per program: residency PD; PC; chair or vice/associate chair; APD; residency core faculty member; general faculty member. Free-text responses were coded using a thematic analysis between two authors (BM, MKu) for the SOAP qualitative data, and by two authors (BM, JM) for the interview uniformity, decreasing applicants, and future directions qualitative data. Simultaneous coding was allowed. Any disagreements between codes were resolved by two other authors (AS, MK), and if no agreement could be reached the response was not analyzed. For all questions, only three responses were discarded due to not being able to reach an agreement.

## RESULTS

### Filled and Unfilled Program Data

In total, 245 responses to the survey were recorded. Twelve (4.9%) were excluded due to incomplete responses, and 74 were discarded due to either duplicate responses or multiple responses being submitted from different representatives from the same residency program. There were responses from 160 of the 287 EM residency programs that exist nationally, representing a 55.7% response rate. Respondents from the programs included 109 PDs (67.7%), 33 APDs (20.5%), seven CDs (4.3%), six faculty members (3.7%), five PCs (3.1%), and one vice chair (0.7%). We compared demographic information of responding programs to all known EM programs based on the American Medical Association (AMA) Fellowship and Residency Electronic Interactive Database and the American Board of Emergency Medicine data, which is presented in Table 2. ^11^ ^,^ ^12^

**Table 2.** Demographic information comparing all US emergency medicine programs to those that responded to the Council of Residency Directors Match Task Force survey regarding the 2023 match.

	All EM programs (N=287)	All responding programs (n=160)	Filled responding programs (n=101)	Unfilled responding programs (n=59)
Region				
Northeastern	86 (29.9%)	50 (31.3%)	27 (26.7%)	23 (39%)
Southern	91 (31.5%)	42 (26.3%)	31 (30.7%)	11 (18.6%)
Central	70 (24.3%)	39 (24.4%)	20 (19.8%)	19 (32.2%)
Western	41 (14.2%)	29 (18.1%)	23 (22.8%)	6 (10.2%)
Hospital setting				
Academic/university	97 (33.8%)	64 (40%)	50 (49.5%)	14 (23.7%)
Community	55 (19.2%)	41 (25.6%)	14 (13.9%)	27 (45.8%)
Hybrid	130 (45.2%)	44 (27.5%)	28 (27.7%)	16 (27.1%)
County		11 (6.9%)	9 (8.9%)	2 (3.4%)
Other (military, etc)	5 (1.7%)			
Training format				
PGY 1–3	233 (81.2%)	125 (78.1%)	78 (77.2%)	47 (79.7%)
PGY 1–4	54 (18.8%)	35 (21.9%)	23 (22.8%)	12 (20.3%)
Age of program				
<5 years	86 (29.9%)	24 (15%)	12 (11.9%)	12 (20.3%)
5–10 years	46 (16%)	23 (14.3%)	12 (11.9%)	11 (18.6%)
11–20 years	34 (11.8%)	25 (15.6%)	17 (16.8%)	8 (13.6%)
>20 years	121 (42.1%)	85 (53.1%)	57 (56.4%)	28 (47.5%)
Unsure	NA	3 (1.9%)	3 (2.9%)	0

*EM*, emergency medicine; *PGY*, postgraduate year.

On average, program leaders reported interviewing 14.9 applicants per position (SD 4.76) in the 2022–2023 application cycle. Compared to the 2021–2022 application cycle, programs reported interviewing 18.7 more applicants total (range: −105 to +185, SD 40.16). Regarding creation of a rank order list (ROL), programs indicated that they placed a mean of 13.9 applicants on their ROL per position (SD 4.39). Compared to the prior application cycle, programs placed a mean of 15.6 more applicants on their ROL (SD 28.4). There were no statistically significant differences between filled and unfilled programs in terms of number of applicants interviewed per residency position (*P* = 0.37) or number of applicants on the ROL per position (*P* = 0.55), using two-tailed *t*-tests.

Compared to the 2021–2022 recruitment season, 46/131 respondents (35.1%) indicated that they made no significant changes in the consideration of the formation of their ROL, and 47% indicated that they included applicants with less desirable Standard Letters of Evaluation (SLOE) compared to prior years. Additionally, 39.7% indicated they included those with more “red flags” on their applications, such as standardized test failures, remediation of clerkships, or professionalism issues. A similar 39.7% indicated that they ranked applicants with a lower class rank compared to years prior, while 18.3% responded that they ranked more individuals with less leadership or volunteerism, and 12.9% indicated that they ranked more of those who they felt did not align with the mission or values of the program. Five programs (3.8%) stated that they considered more osteopathic applicants, and another five (3.8%) indicated that they considered more international medical graduates (IMG).

While preference signaling was new to EM this year, it has been used by other specialties, such as otolaryngology, since the 2020–2021 application cycle.^13^ Emergency medicine programs had varying ways in which they used preference signaling during this application cycle. Table 3 provides details of how programs interpreted signal preferencing.

**Table 3.** How residency programs used preference signaling.

More likely to interview applicants that signaled	32 (23.7%)
Minor change to interview selection process	24 (17.8%)
No change to interview selection process	24 (17.8%)
Interviewed most but not all applicants that signaled	13 (9.6%)
Interviewed all applicants that signaled	12 (8.9%)
Signal was used as a tiebreaker between similar applicants	12 (8.9%)
Signal was considered when inviting applicants from the waitlist	7 (5.2%)
Signal was used for out-of-region applicants	6 (4.4%)
Did not opt in	5 (3.7%)

More than half of respondents felt applicant quality was either a little worse this year (9.7%) or substantially worse this year (42.5%). A minority (6.7%) felt applicant quality had improved this year. Perceptions of Match results were similar to perceptions of applicant quality. More than half felt their program’s Match results were a little worse (39.4%) or substantially worse (19.7%) than the previous year. Notably, 11.4% felt their Match results were better than the previous year, and 30% indicated similar Match result quality to the prior year. A majority of programs indicated that they went lower down their rank list, with 75.2% indicating that they either went a little deeper or substantially deeper compared to prior years.

### Unfilled Program and SOAP Data

Of the 132 unfilled programs, 59 (44.7%) of their program leaders responded to this survey. On average, programs had 4.8 positions unfilled (range 1–13, SD 2.87) out of an average cohort size of 10 residents per class (range 6–16, SD 3.42), yielding a mean vacancy rate per unfilled program of 47.8%. Of the responding programs that did not fill in the 2023 Match, 40.7% did not fill in the 2021–2022 application cycle. Program leaders reported receiving an average of 257 total SOAP applications (SD 130), or 53.6 applications per unfilled spot. Programs reported interviewing an average of 16.2 applicants per unfilled position in their program (range 5.8–40, SD 9.68); 83.3% of programs reported they were able to fill all unfilled positions in the SOAP. Table 4 outlines program perspectives on the underlying reasons why they felt their program did not fill in the Match.

**Table 4.** Top factors that programs believed contributed to not filling in the 2023 match.

Workforce concerns	39 (76.5%)
Geographic location of program	30 (58.8%)
Increasing number of EM spots	24 (47.1%)
Virtual interviews format	23 (45.1%)
New program	8 (15.7%)
Sponsoring institution (university vs CMG)	7 (13.7%)
Program specific factors (wellness, curriculum changes, etc)	6 (11.8%)
Social media issues	4 (7.8%)
New leadership	3 (5.9%)
Accreditation status	2 (3.9%)

*EM*, emergency medicine; *CMG*, contract management group.

Regarding applicants in the SOAP and their preparedness to practice EM, 35 leaders of unfilled programs gave information about their applicants. Eighteen (51.4%) stated that most applicants had completed at least one EM rotation but noted that it was after the time that ERAS applications were due, leading to late consideration of EM as their desired medical specialty. Five respondents (14.3%) reported most applicants had completed one EM rotation but mentioned no details about the timing of that rotation. Only two respondents (5.7%) reported that the typical applicant had no or minimal exposure to EM. Interestingly, 20% of program leaders mentioned that many applicants had exposure to EM prior to starting medical school, such as working as a scribe or paramedic. Program leaders also reported that roughly 15% of applicants had at least one EM SLOE available for them to review.

Program leaders reported they were relatively content with applicants available to them in the SOAP, with 78% responding that they were either extremely or somewhat satisfied with the quality of applicants. In addition, 80% reported that SOAP applicants were either significantly or slightly better compared to the bottom quartile of their original ROL.

Program leaders were also asked what worked well regarding the SOAP process itself. Free-text responses underwent thematic analysis as described above with 37 recorded responses evaluated. Eleven (29.7%) stated that it was an opportunity for collaboration within their program leadership and faculty group. Ten (27%) mentioned that they thought their pre-planning strategy and organization during the SOAP worked well. Four respondents (10.8%) explicitly mentioned that the NRMP and Electronic Residency Application Service technology worked well. Additionally, 8.1% mentioned the strong quality of SOAP applicants available to them, 5.4% of respondents noted adequate support from CORD, and another 5.4% noted there was enough time to navigate the SOAP and interview applicants.

Conversely, program leaders were also asked about the challenges they faced during the SOAP, with 49 responses included in the following analysis. Twelve (34.7%) thought there were too many applicants and not enough time to review their applications and interview them. Ten (20.4%) disliked the format of SOAP offers, noting their desire for either additional rounds or that programs should be able to offer spots to more candidates. Six (12.2%) noted difficulties with disingenuity from applicants or violating NRMP SOAP rules. Three programs (6.1%) noted a lack of qualified applicants, while two (4.1%) noted concern over the applicant’s interest in a career in EM. Finally, three programs (6.1%) responded that there were issues with the overall number of unfilled programs and competition between programs for SOAP candidates.

### Qualitative Data About the Future of EM and Next Steps

The survey asked open-ended questions about standardization of the interview process: 41.2% of respondents indicated they would like to have a mandated return to in-person interviews, while 11.8% preferred a requirement for virtual interviews. Overall, 13.7% wanted interview uniformity among programs, and 3.9% voiced a desire for flexibility to allow programs to do what worked for them. Additionally, 9.8% stated they would like to have uniform cancellation standards for applicants. When asked directly, 74.3% responded reported they would like to see an interview cap enforcement. Of the 94 respondents who supported an interview cap for applicants, the mean suggested cap was 17.3 interviews per applicant (range 6–50, SD 6.7).

Program leaders were also asked why they thought fewer medical students were applying to EM. The most common response was that applicants were concerned about the future of the workforce, which 78.1% of respondents listed as a top concern. Further results for this question are listed in Table 5. Finally, program leaders were also asked what actions they would like to see taken to help support the future of training in EM. Results are shown in Table 6.

**Table 5.** Reasons why program leaders believe fewer students are applying to emergency medicine.

Workforce concerns	107 (78.1%)
Burnout	46 (33.6%)
Work environment	42 (30.7%)
COVID-19/pandemic	37 (27%)
Boarding	36 (26.8%)
Corporatization of EM	21 (15.3%)
Negative EP modeling	20 (14.6%)
Negative press	18 (13.1%)
Advising	14 (10.2%)
Lack of early exposure to EM	10 (7.3%)
Increased roles of non-physician practitioners	6 (4.4%)
Salary	5 (3.6%)

*EM*, emergency medicine; *EP*, emergency physician.

**Table 6.** Actions residency program leaders would like to see to help support the future of emergency medicine.

Halt opening of additional EM programs	32 (25%)
Increase RRC standards for EM	28 (21.2%)
Decrease number of programs	24 (18.8%)
Decrease number of total EM spots	24 (18.8%)
Positive messaging campaign	22 (17.2%)
Counter workforce study	16 (12.5%)
Close CMG-sponsored programs	8 (6.25%)
Mandate 4-year programs	5 (3.9%)
Improve work environment	4 (3.1%)
Applicant resources for finding program best fit	2 (1.5%)
Increase early exposure to EM	2 (1.5%)
Produce a “rating system” of EM programs	1 (0.8%)
Expand scope of EM	1 (0.8%)
Combat non-physician practitioner scope expansion	1 (0.8%)
Increase resources for international medical graduates	1 (0.8%)

*EM*, emergency medicine; *RRC*, Residency Review Committee; *CMG*, contract management group.

## DISCUSSION

This study builds upon the work that was started by Murano et al following the 2022 Match. Here, we describe factors that educational leaders believe contributed to the decreasing number of applications to EM and to the increasing number of both unfilled programs and open residency positions. Results of this study are consistent with previous studies identifying geography, specifically location in the Northeastern and Central United States, as a characteristic of unfilled programs.^14^ In fact, 58.8% of unfilled program leaders in this study believed geographic location was a major contributing factor to their program not filling in the 2023 Match. Another important factor identified by unfilled program leaders was the increasing number of EM spots. There were no statistically significant differences in the number of applicants interviewed per position, or number of applicants placed on the ROL, by filled compared to unfilled programs. Therefore, widespread interviewing and ranking of more applicants by EM programs would likely not be helpful in improving the overall Match results because of the declining applicant pool and excess of training spots.

Virtual interview format has been supported by CORD since the beginning of the COVID-19 pandemic.^15^ While this may help to decrease costs associated with residency interviews for applicants, lessen the carbon footprint associated with travel for interviews, and increase the amount of time available to focus on clinical exposure in medical school, it may also lead to students applying to and interviewing with more programs.^16^ In the 2023 Match, students applying to EM applied to a median of 69 programs and interviewed at a median of 18.5 programs according to NRMP Charting Outcomes.^17^ Comparatively, in 2019 the average US graduate applicant applied to 57 programs.^18^ This increased number of applications makes it very difficult for program leaders to know which applicants are truly interested in their program vs those who applied and interviewed due to the ease of interviewing virtually. This sentiment is supported by the results of this survey, with 45.1% of unfilled program leaders stating that virtual interviews were a key contributor to why they were unfilled, and 41.2% of respondents voicing a desire to return to in-person only interviews, compared to only 11% who want to continue a virtual-only interview format.

Furthermore, a majority of program leaders were in favor of capping interviews (73.4%), with a mean suggested cap of 17 interviews. However, it is not currently known whether an interview cap is permissible or enforceable through the NRMP. Neither is it known whether an interview cap would disproportionately harm certain programs, such as more rural, smaller, or traditionally less competitive programs. Interview caps, however, have been used in other specialties. In response to virtual interviews and the COVID-19 pandemic, ophthalmology has employed interview caps for their match since the 2020–2021 application cycle and, in fact, just lowered the cap of interviews from 18 to 15.^19^ Obstetrics and gynecology is also considering implementing an interview cap and, in a simulated environment, found that it increased the odds that less-competitive applicants would be offered interviews.^20^

Preference signaling was implemented for the first time in 2023 for EM, which was reported as a largely desired change in the Murano et al study. Programs used these signals in a variety of ways; however, the plurality of programs stated that receiving a signal made them more likely to offer an interview. Additionally, 17.8% relayed that it made no difference on the decision to interview, and only 3.7% of responding program leaders did not opt in to receive preference signals. Changes to preference signaling for the 2023–2024 match, including the increase from five to seven signals and the introduction of geographic preference signaling may affect how applicants and programs use signaling. Future research will be needed on preference signaling as it evolves to include geographic region signals instead of signals targeted at individual programs alone.

Other groups, such as the Emergency Medicine Resident Association, have speculated as to why fewer medical students are choosing EM as their intended specialty, with workforce projections, concern for increasing scope given to non-physician practitioners, and burnout topping the list.^21^ The results of this study, which could be considered as consensus expert opinion, are in agreement with several of those speculations, with over 75% of program leadership believing concern for an oversupply of EPs is the leading cause of declining application numbers. Other top contributors from this survey include burnout, which according to the most recent AMA survey, places EM as the specialty with the highest rate of burnout, with 62% of EPs reporting burnout.^22^ This degree of burnout and concern over the workforce likely contributes to why other respondents believed negative EP modeling (14.6%) and advising from EPs and medical school deans (10.2%) contributes to fewer students choosing EM. It is also important to note that a difficult work environment (lack of needed resources, nursing and drug shortages, difficult interactions with admitting teams and consultants), in-patient boarding in the ED, and the long-lasting stress that COVID-19 and concern for future novel infectious diseases are also top reasons why education leaders believe fewer students are choosing EM.

Frequently, the situation of EM today is compared to the expansion of residency positions in anesthesiology in the 1980s and 1990s. During that time, residency spots nearly quadrupled, until concern about oversupply of anesthesiologists caused decreased applications to the specialty and eventual contraction of the number of spots.^23^ ^,^ ^24^ Similarly, decreasing the number of EM trainee spots was a key theme for respondents when questioned about what actions they believed should be taken to address the increasing number of unfilled EM positions: 25% suggested not allowing any new programs to open; and 37.6% wanted to decrease either the number of overall programs or the number of residency positions. Many (21.2%) expressed the belief that increasing the Accreditation Council for Graduate Medical Education Residency Review Committee standards for EM is a way to accomplish this.

In addition to halting growth of EM residency programs and decreasing the overall number of EM trainees, respondents also voiced a desire to begin a positive messaging campaign about EM and its future, which CORD has already begun on social media.^25^ Another suggested action was to counter the American College of Emergency Physicians 2030 workforce study (12.5%), which several others have already done, mainly citing a low attrition rate in the original study.^26^ Lastly, it is important to note that the 2024 Match results for EM yielded fewer open spots and fewer unfilled programs compared to 2023. The CORD EM Match Task Force has ongoing work to determine how programs changed their recruitment strategies and how this could have affected the Match results, or whether this truly represents an improvement in the Match environment for EM.

## LIMITATIONS

This was a voluntary survey subject to selection bias, as those with strong needs and opinions were more likely to complete the survey. In addition, because this survey was distributed both at the CORD Academic Assembly and through the CORD PD Listserv, sampling was limited to those programs involved within this organization. However, a 55.7% response rate from all EM programs suggests that this dataset represents a broad array of programs and ideas. Data collection began in March 2023 in the weeks immediately following the NRMP Match and SOAP. While this helped to increase the response rate and added to data validity, it may have made many of the free-text responses regarding actions that should be taken more emotionally charged.

Finally, this paper presents the opinions and voices of educational leaders in EM and may not represent the reality of the applicant pool to EM residency or the future of training in EM. Results reported here should not be taken as advice from CORD or from the EM Match Task Force.

## CONCLUSION

Here we present data regarding the 2023 Match environment for EM and describe a residency program-level needs assessment and desire for action. Most program leaders believed that the decreasing number of EM applicants was due to concern over the EM workforce, burnout in EM, and difficulties with the work environment. A majority were in favor of interview caps. Program leaders also voiced a desire for overall fewer training spots in EM, among several other ideas. Annual review of the Match data and residency program needs should be continued until improvement occurs in the Match environment for EM.
